# What good are positive emotions for treatment? A replication test of whether trait positive emotionality predicts response to exposure therapy for social anxiety disorder

**DOI:** 10.1016/j.brat.2023.104436

**Published:** 2023-11-11

**Authors:** Charles T. Taylor, David Rosenfield, Sheila M. Dowd, Christina D. Dutcher, Stefan G. Hofmann, Michael W. Otto, Mark H. Pollack, Jasper A.J. Smits

**Affiliations:** a Department of Psychiatry, University of California, 9500 Gilman Drive (Mail code 0855) La Jolla, San Diego, CA, 92093-0855, United States; b Department of Psychology, Southern Methodist University, United States; c Department of Psychiatry & Behavioral Sciences, Rush University Medical Center, United States; d Department of Psychology and Institute for Mental Health Research, University of Texas at Austin, United States; e Department of Clinical Psychology, Philipps-University Marburg, Germany; f Department of Psychological and Brain Sciences, Boston University, United States; g Sage Therapeutics, United States

**Keywords:** Social anxiety disorder, Positive emotion, Exposure therapy, Randomized controlled trial, Prediction

## Abstract

**Background:**

Positive valence emotions serve functions that may facilitate response to exposure therapy – they encourage approach behavior, diminish perceived threat reactivity, and enhance assimilation of new information in memory. Few studies have examined whether positive emotions predict exposure therapy success and extant findings are mixed.

**Methods:**

We conducted a secondary analysis of an exposure therapy trial for social anxiety disorder to test the hypothesis that patients endorsing higher trait positive emotions at baseline would display the greatest treatment response. N = 152 participants enrolled in a randomized controlled trial of d-cycloserine augmentation completed five sessions of group exposure therapy. Pre-treatment positive emotionality was assessed using the NEO Five-Factor Inventory. Social anxiety symptoms were assessed throughout treatment by blinded evaluators using the Liebowitz Social Anxiety Scale.

**Results:**

Accounting for baseline symptom severity, multilevel growth curve models revealed that patients with higher pre-treatment positive emotionality displayed faster social anxiety symptom reductions and lower scores at 3-month follow-up. This predictive effect remained significant after controlling for baseline depression and extraversion (without the positive emotionality facet).

**Conclusions:**

These findings add to emerging evidence suggesting that explicitly targeting and enhancing positive emotions during exposure to perceived threat may improve treatment outcomes for anxiety and fear-based disorders.

**Trial registration:**

ClinicalTrials.gov: NCT02066792 https://clinicaltrials.gov/ct2/show/NCT02066792

Exposure therapy is the core component of first-line cognitive and behavioral treatments for anxiety and fear-based disorders. It is based on the premise that recovery from anxiety occurs when a person repeatedly confronts threat-relevant cues or contexts and learns that feared stimuli are not as dangerous as previously thought (McNally, 2007; Smits, Powers, & Otto, 2019). Such learning requires one to approach and engage with avoided feared situations, tolerate unpleasant experiences (e.g., distress), and assimilate new, threat-inconsistent information into memory (Craske et al., 2008). Because exposure therapy response rates approximate 50% (Loerinc et al., 2015), there is a need to identify theory-driven and modifiable predictors of treatment success that could be leveraged to improve treatment outcomes. Here, we conducted a secondary analysis of an exposure therapy trial for social anxiety disorder (SAD) to examine whether individual differences in the trait-like tendency to experience positive valence emotions predicts treatment success.

Affective science findings suggest positive valence emotions (e.g., joy, interest, contentment) and their associated response tendencies (e. g., behavioral approach) serve functions that support adaptive responses to perceived threat and promote new, non-threat learning. Individual differences in positive affect (i.e., the frequency and intensity with which a person experiences positive valence emotions) as well as experimentally inducing positive emotions relative to neutral or negative emotions (e.g., sadness) in non-clinical samples: (1) downregulates physiological (Fredrickson, Mancuso, Branigan, & Tugade, 2000) and subjective reactivity (e.g., negative affect) (Speer & Delgado, 2017) to perceived threat; (2) facilitates tolerance of aversive experiences (de Wied & Verbaten, 2001); (3) activates approach motivation and behavior, including engagement with threat-relevant exposures (Berman, Summers, Weingarden, & Wilhelm, 2019); (4) promotes adaptive coping (e.g., positive reappraisal) in stressful situations (Tugade & Fredrickson, 2004); and (5) increases awareness and assimilation of new information, including promoting openness to new information and patterns of information processing (see review by (Fredrickson, 2013)). Positive affect has also been shown to facilitate mechanisms supporting learning and memory, including enhancing encoding, rehearsal, and retrieval (see review by (Zbozinek & Craske, 2017b)) – processes that underpin extinction learning (Craske et al., 2008; Kredlow, Eichenbaum, & Otto, 2018).

Data from fear conditioning and extinction paradigms in healthy samples – an experimental analogue of exposure therapy – suggests positive affect may inhibit the return of fear following extinction training (Zbozinek & Craske, 2017a; Zbozinek, Holmes, & Craske, 2015). In a cross-sectional study, higher positive affect (but not negative affect) before and after extinction was associated with less return of fear during reacquisition [i.e., re-pairing of the CS+ and US following extinction] as measured by skin conductance arousal and fear expectancy (Zbozinek & Craske, 2017a). Experimentally inducing positive affect prior to extinction training decreased subsequent negative valence appraisals of the conditioned aversive stimuli and led to less return of fear during reinstatement [i.e., exposure to the US in the absence of the CS+] one week later (Zbozinek et al., 2015). Conversely, anhedonia (a clinical phenotype characterized by low positive affect), but not general distress or fears, was associated with increased activity in threat-related neural circuity (e.g., amygdala, anterior insula) in response to an extinguished threat stimulus in a cross-section of young adults (Young et al., 2021). Those findings point to a persistence of inflated threat reactivity when danger is no longer present in people with low positive affect – complementing extinction training studies linking positive affect and extinction learning in healthy samples. Research in patient samples further supports the positive emotionality-threat reactivity link: adults with SAD characterized by higher positive affect experienced lower anticipatory anxiety and displayed less anxiety-related behavior in response to a standardized public speaking exposure, beyond level of negative affect (Taylor, Tsai, & Smith, 2020).

Findings from laboratory-based studies suggest individual differences in positive emotions may predict response to exposure-based therapies for anxiety. Initial support for that hypothesis came from a secondary analysis of an exposure-based CBT trial for patients diagnosed with panic disorder or generalized anxiety disorder (Taylor, Knapp, et al., 2017). Higher pre-treatment levels of trait positive emotionality (measured using the positive emotion facet of the Revised NEO Personality Inventory) predicted greater reduction in anxiety symptoms and fewer symptoms following treatment, even when accounting for baseline levels of depression and disorder-specific symptom severity. Responder status was greater in participants who scored above the normative sample mean on positive emotionality vs. those who scored below (71% vs. 40%). However, a subsequent study in patients diagnosed with SAD did not find that baseline levels of positive affect predicted response to CBT or acceptance and commitment therapy (Sewart et al., 2019). In this study, positive affect was measured in reference to the past week using a composite of the Positive and Negative Affect Schedule and Mood and Anxiety Symptom Questionnaire positive affect items (cf. using a personality assessment inventory; (Taylor, Knapp, et al., 2017)), and hence was more of a “state” measure of positive affect, not a “trait” measure. Similarly, state positive emotional reactivity in response to viewing positive valence images did not predict response to CBT or ACT for SAD (Niles, Mesri, Burklund, Lieberman, & Craske, 2013). Thus, despite laboratory evidence suggesting positive emotions may support processes believed to underpin exposure therapy success, prediction of clinical response from pre-treatment measures of positive emotions is mixed.

To further investigate the possibility that positive emotions are relevant for exposure therapy success, we sought to replicate and extend to a sample of patients with SAD prior findings (Taylor, Knapp, et al., 2017) demonstrating that higher pre-treatment positive emotionality predicted superior clinical response. The replication sample comprised patients enrolled in a d-cycloserine (DCS) exposure augmentation trial for SAD in which all patients were instructed to repeatedly confront feared social situations (e.g., public speaking exposures; ClinicalTrials.gov Identifier: NCT02066792) (Hofmann et al., 2015; Smits et al., 2020). Similar to (Taylor et al., 2017a), participants completed a pre-treatment measure of positivity emotionality derived from the NEO Five-Factor Inventory of personality. Clinician-rated social anxiety symptoms were assessed before, during (weekly), and after treatment. Linear mixed effect models tested the hypothesis that higher pre-treatment positive emotionality would be associated with a larger reduction in symptoms (H1) and lower symptom severity at the final follow-up (H2). Because depression frequently co-occurs with social anxiety (Kessler, Chiu, Demler, Merikangas, & Walters, 2005) and is also characterized by low positive affect (Brown, Chorpita, & Barlow, 1998), secondary models also controlled for baseline depression scores. Finally, to account for the possibility that the higher order extraversion domain, rather than positive emotionality per se, would predict treatment response (Taylor, Knapp, et al., 2017), we examined a model also controlling for baseline extraversion scores that did not include the positive emotion facet.

## 1. Method

### 1.1. Participants

Participants were enrolled across three sites between February 2015 and January 2018. Inclusion criteria were: age 18–70 (inclusive); diagnosis of SAD according to the Diagnostic and Statistical Manual of Mental Disorders (Fifth Edition) (DSM-5) criteria; Liebowitz Social Anxiety Scale (LSAS) ≥ 60. Exclusion criteria were lifetime history of bipolar, psychotic, or obsessive-compulsive disorder; eating disorder, posttraumatic stress disorder, or substance use disorder in the past 6 months; any potentially interfering cognitive dysfunction; significant suicidal ideation or suicidal behaviors in the past 6 months; serious medical illness; history of seizures; pregnancy, lactation, or of child-bearing potential and not using contraception; or concurrent psychotherapy or pharmacotherapy or prior nonresponse to exposure therapy. The analysis sample included participants who were randomized to one of four treatment arms (N = 152). Sample demographics were: age (*M* = 29.24, *SD* = 10.16), sex (84 women [55.26%], 67 men [44.08%] women, 1 neither [0.66%]), race (90 [59.21%] White, 34 [22.37%] Asian, 20 [13.16%] Black, 5 [3.29%] other, and 6 [3.95%] not reported), ethnicity (30 [19.74%] Hispanic or Latino, 116 [76.32%] not Hispanic or Latino, 6 [3.95%] not reported).

### 1.2. Measures

#### 1.2.1. Positive emotions

Trait positive emotions were assessed using the NEO Five-Factor Inventory (NEO-FFI; (Costa & McCrae, 1992)). The NEO FFI is a 60-item self-rated measure designed to assess five personality domains: neuroticism, extraversion, agreeableness, conscientiousness, and openness to experience. Facets within each domain describe lower-level groups of personality characteristics. Research supports a 4-item positive emotions scale within the domain of extraversion (items: laughs easily; not cheerful or light-hearted [R]; cheerful, vivacious; not a cheerful optimist [R]) (Chapman, 2007; Saucier, 1998). The NEO FFI positive emotion scale is reliable and valid, and correlates highly (r = 0.86) with the positive emotion facet score of the 240-item NEO-PI-R (used to predict treatment response in (Taylor, Knapp, et al., 2017)). Current sample Cronbach’s alpha = 0.76. The remaining NEO FFI extraversion items (reflecting sociability and activity) were summed (without the positive emotion items; Cronbach’s alpha = .70 and entered into secondary analysis models (described below).

#### 1.2.2. Depression

The Montgomery–Åsberg Depression Rating Scale (MADRS; (Montgomery & Asberg, 1979)) was used to assess depression severity at baseline. It comprises 10 items rated by clinicians on a seven-point Likert scale. Items are summed to produce a total scale score ranging from 0 to 60, with higher scores reflecting greater depression severity.

#### 1.2.3. Social anxiety symptom severity

Trained evaluators blind to participant’s treatment condition administered the LSAS (Liebowitz, 1987) each week to measure severity of social anxiety symptoms. Participants rated their level of fear and avoidance within the past week for 24 social or performance situations on a 4-point scale ranging from 0 (no fear/never avoids) to 3 (severe fear/usually avoids). LSAS was assessed weekly during treatment and at the 1-month and 3-month follow-ups.

### 1.3. Procedure

Potential participants were invited for a full in-person eligibility assessment after providing informed written consent. Eligible participants met with an independent evaluator who administered the baseline outcome measures. The following week, 160 of 169 participants attended the first session (60 min) of a 5-session group exposure therapy program (Hofmann et al., 2015), which provided education on SAD and a rationale for exposure therapy. The remaining sessions (2–5; 90 min) focused on public speaking practice designed to evoke adequate fear activation and provide opportunities for violating threat expectancies. Between session exposure practice was encouraged.

Randomization occurred at the beginning of session 2 (N = 152) in a double-blind fashion to one of four DCS/placebo augmentation regimens based on the timing (before or after) and success of the exposure session (success was defined as achieving an end of exposure fear score of 40 or less on a scale of 0–100). Treatment conditions were: (1) placebo before the session and either DCS after a successful session or placebo after an unsuccessful session (tailored); (2) DCS before the session and placebo after the session (pre-session); (3) placebo before the session and DCS after the session (post-session); (4) placebo before and after the session (placebo). Study medication was administered and monitored by research staff blind to treatment condition.

### 1.4. Statistical analyses

The growth curve model of LSAS over the course of the study was analyzed using mixed effects models (MEMs). MEMs include all participants who have at least one assessment, and can model the complex covariance of the repeated measures over time. We first determined the best fitting growth curve model, comparing linear, quadratic, and log growth models and choosing the model with the lowest Bayesian Information Criterion [BIC]. Then we determined the best fitting covariance structure of the error covariance matrix, comparing diagonal, AR (1), compound symmetry, Toeplitz, and unstructured covariance structures, and random effects models, again choosing the model with the lowest BIC (please see the Supplement for the definition of each of these covariance structures).

As noted above, the parent study was comprised of 4 different DCS treatment groups. Although all 4 DCS treatment groups received the same exposure treatment (just different DCS protocols), participants responded better in some treatment groups than in others. To ensure that the obtained effects of Positive Emotionality were not due to DCS treatment group difference (even though there were no treatment group differences in Positive Emotionality, p = .657), all analyses controlled for DCS Treatment Group and the DCS Treatment Group × Time interaction.

Because Positive Emotionality was related to baseline LSAS severity (higher Positive Emotionality was related to lower baseline LSAS, *r* (150) = −0.28, *p* < .001), all models also controlled for baseline LSAS and baseline LSAS × Time. Otherwise, the beneficial effect of Positive Emotionality might be due to lower baseline LSAS severity. Thus, all models included Positive Emotionality, Time, Positive Emotionality × Time, baseline LSAS, baseline LSAS × Time, DCS Treatment Group (dummy coded), and DCS Treatment Group × Time. The growth curve model tracked LSAS from the beginning of the first session of exposure treatment through the final assessment (3-month follow-up). In sensitivity analyses, we reran all analyses without controlling for baseline LSAS and DCS treatment groups to determine if the results for Positive Emotionality were due to inclusion of these control variables.

We also examined whether treatment responders (defined as LSAS < 50 at follow-up, as per Pollack et al., 2014) had higher baseline Positive Emotionality scores than non-responders. Following the approach used in our previous paper on this topic (Taylor, Lyubomirsky, & Stein, 2017), we included all participants in this analysis by employing our intent-to-treat MLM models to estimate the follow-up scores of participants who did not complete the follow-up assessment. We then performed a oneway ANOVA, with the independent variable being responder status (yes/no), and the dependent variable being baseline Positive Emotionality score.

Sensitivity analyses examined whether the effects of Positive Emotionality varied between DCS treatment groups by including the interactions between the dummy variables coding the 4 DCS treatment groups and the Positive Emotionality × Time interaction. Exploratory analyses investigated whether the effect of Positive Emotionality on the effectiveness of the exposure treatment remained after controlling for baseline depression by adding baseline MADRS scores, and the baseline MADRS × Time interaction, to the primary model above. In a second exploratory analysis, we controlled for baseline extraversion by adding baseline extraversion (minus the Positive Emotionality items) and the baseline extraversion × Time interaction to our primary model. Finally, in a third exploratory analysis, we controlled for both baseline MADRS and extraversion by adding baseline MADRS and extraversion (minus the Positive Emotionality items), and their interactions with Time, to our primary model.

Effects were considered significant if p < .05. Approximate effect sizes for all significant effects were calculated using the *t*-to-*d* (Cohen’s d) conversion. The BIC for each model is reported below. Although one cannot statistically compare models based on the BIC, Singer and Willett (2003) suggest that differences between models of 0–2 points is “weak”, 2–6 is “positive”, 6–10 is “strong”, and over 10 is “very strong.”

## 2. Results

### 2.1. Initial analyses

The CONSORT diagram for the parent trial (Smits et al., 2020) is included in the supplement (Supplemental eFig. 1). Box plots and strip plots for the variables of interest in the present study are shown in the Supplement in eFig. 2. Scatterplots between these variables are shown in the Supplement eFig. 3. As reported in the parent trial, the study included 152 adults with SAD (mean[SD] age, 29.24[10.16] years, 84 [55.26%] of which were male). There were no differences between DCS Treatment Groups on demographic variables or outcome measures at baseline (supplement eTable 1). Session attendance was high (N = 139 (91.4%) attended through the last exposure session). Attrition at the 3-month follow-up was N = 29 of 152 [19.1%]. Attrition did not differ between groups. Participants’ demographic and baseline measures were not different between those with missing data vs. those with complete data. The primary trial reported the differences between the 4 DCS groups so those differences will not be reported here unless there is an interaction between the effects of Positive Emotionality and the DCS treatment groups.

### 2.2. Positive emotions as a predictor of treatment outcome

The best fitting growth curve model for the change in LSAS over time (lowest BIC) was a linear model coding assessment number, from assessment 1 (beginning of the first exposure session) to assessment 8 (the 3-month follow-up). Time was then centered at assessment 8 so that the main effect of Positive Emotionality would reflect the effect of Positive Emotionality on LSAS at assessment 8 (the 3-month follow-up). The best fitting covariance structure (lowest BIC) for the errors of the repeated measures was AR(1). All the findings reported herein were robust (i.e., did not change in significance) to changes in the growth curve model (e.g., using LN of weeks as the Time variable instead of linear assessments) and to changes in the covariance structure (e.g., using a Toeplitz matrix instead of AR(1) for the error covariance).

**Fig. 1.**
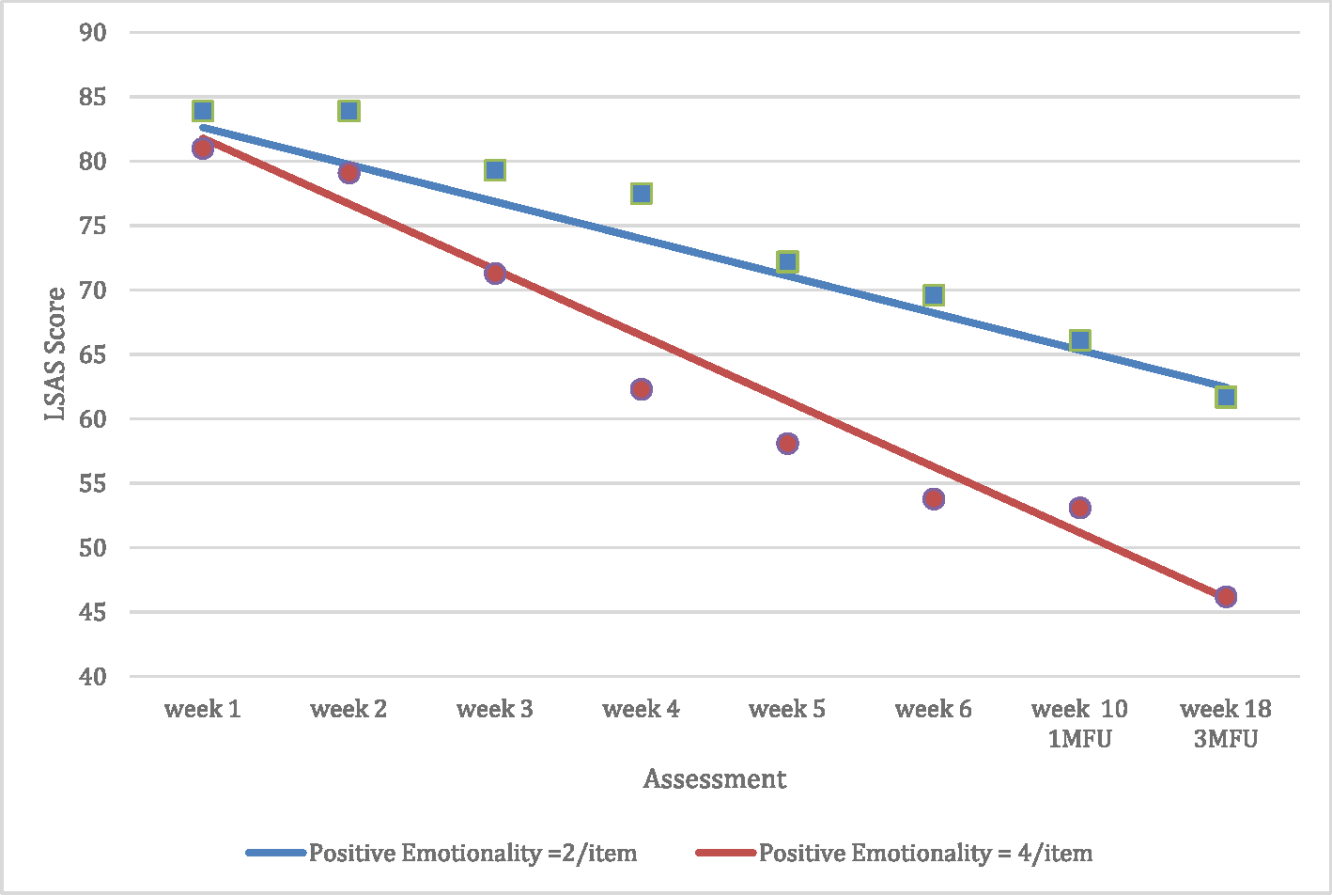
Estimated LSAS scores from baseline through 3-month follow-up at different levels of baseline Positive Emotionality. *Note*: 1MFU = 1-month follow-up; 3MFU = 3-month follow-up. Blue squares indicate the raw means for those with low Positive Emotionality (mean = 2.01/item). Red Circles indicate the raw means for those with high Positive Emotionality (mean = 4.14/item). These estimated means come from analyses in which baseline LSAS and its interaction with Time are controlled. (For interpretation of the references to colour in this figure legend, the reader is referred to the Web version of this article.)

Average Positive Emotionality scores across the 4 Positive Emotionality items ranged from 1.25 to 5.0, with a mean (and median) of 3.0 (SD = 0.82). Consistent with hypothesis 1, our MEM analysis showed that the slope of decrease (improvement) in LSAS from the beginning of exposure sessions to the 3-month follow-up was steeper for participants with higher baseline Positive Emotionality, *b* = −1.11, *95% CI* [−1.70, −0.52], *t*(1102) = − 3.67, *p* < .001, *d* = 0.22 (see Fig. 1; BIC for this model was 8463.97). For example, for participants who averaged a score of 4.0 on the 4 Positive Emotionality items, their slope of improvement was b = −5.10, 95% CI [−5.85, −4.35], t(1102) = −13.32, p < .001, d = 0.80 (i.e., their LSAS scores decreased about 5.1 points per assessment). The decrease for participants who averaged a score of 2.0 on the 4 Positive Emotionality items, though still significant, was 44% lower, *b* = −2.88, *95% CI* [−3.63, −2.13], *t*(1102) = −7.51, *p* < .001, *d* = 0.45. As a result of this difference in slopes of improvement, we found that higher pre-treatment positive emotionality was associated with lower symptom severity at the 3-month follow up (hypothesis 2), *b* = −8.19, *95% CI* [−11.33, −5.05], *t*(1102) = −5.12, *p* < .001, *d* = 0.31. Participants with an average score of 2 on Positive Emotionality items were estimated to have a mean LSAS of 62.5 at 3-month follow-up, while those with an average score of 4 on the Positive Emotionality items were estimated to have a score of 46.1. Spaghetti plots for a subset of the total sample are provided in Supplement eFig. 4. eFig. 4a shows the spaghetti plots for the first 15 participants who were classified as “low Positive Emotionality” (in the bottom 25th percentile) and eFig. 4b shows the spaghetti plots for the first 15 participants classified as “high Positive Emotionality” (in the top 25th percentile).

Sensitivity analyses, dropping the terms involving the control variables (baseline LSAS and DCS treatment condition), revealed identical results in terms of significance. For example, in this sensitivity analysis we found that higher pre-treatment positive emotionality was associated with lower symptom severity at the 3-month follow up, *b* = −11.82, *95% CI* [−15.93, −7.71], *t*(1110) = −5.64, *p* < .001, *d* = 0.34. The BIC for this model was 8570.81, substantially worse than the BIC for the full model above (8463.97).

Another sensitivity analysis provided further support to the effect of Positive Emotionality on exposure treatment success from another perspective. For this sensitivity analysis, we examined whether treatment responders (defined as LSAS< 50 at follow-up, as per Pollack et al., 2014) had higher baseline Positive Emotionality scores than non-responders. Overall, 47.7% of participants met criterion for responders. We then performed a oneway ANOVA, with the independent variable being responder status (yes/no), and the dependent variable being baseline Positive Emotionality score. The analysis indicated that responders had higher average baseline Positive Emotionality scores (M = 3.24, SD = 0.76) than non-responders (M = 2.84, SD = 0.86), *F*(1, 149) = 9.36, *p* = .003.

Further sensitivity analyses, adding the interactions between the DCS Treatment Group dummy variables and the Positive Emotionality × Time interaction (and all subcomponents) to the primary MLM model, showed no indication that the Positive Emotionality × Time interaction (or the Positive Emotionality differences at 3-month follow-up) differed between DCS Treatment groups (*ps* ranged from *p* = .559 to *p* = .926). The BIC for this model was 8505.37, substantially worse than for our primary model above (8463.97).

### 2.3. Exploratory analyses

#### 2.3.1. Baseline depression severity

Baseline depression scores on the MADRS, and their interaction with Time, were added to our primary model above to determine if the effects of baseline Positive Emotionality remained significant after controlling for baseline depression. Analyses (which controlled for baseline LSAS and baseline Positive Emotionality, as well as DCS treatment condition) showed that baseline depression significantly moderated the change in LSAS over time, with participants who were higher on baseline depression improving less than those who were lower, *b* = 0.09, *95% CI* [0.05, 0.14], *t*(1089) = 4.00, *p* < .001, *d* = 0.24. The BIC for this model was 8362.21, substantially better than the BIC for the primary model without MADRS (8463.97), indicating that adding baseline MADRS to the primary model substantially improved model fit. This is consistent with the significant effects of both MADRS and Positive Emotionality. Similarly, participants with higher baseline depression had higher LSAS scores at the 3-month follow-up, *b* = .60, *95% CI* [0.39, 0.81], *t*(1089) = 5.63, *p* < .001, *d* = 0.34. See Supplement eFig. 5. Despite this relation, the effects of baseline Positive Emotionality remained significant, and decreased only about 10%, *b* = −1.01, *95% CI* [−1.60, −0.43], *t*(1089) = −3.42, *p* < .001, *d* = 0.21, for the baseline Positive Emotionality × Time interaction, and *b* = −7.54, *95% CI* [−10.58, −4.49], *t*(1089) = −4.85, *p* < .001, *d* = 0.29, for the main effect of baseline Positive Emotionality on LSAS symptoms at the 3-month follow-up.

### 2.3.2. Extraversion (without positive emotionality items)

Next, baseline extraversion scores (excluding the 4 Positive Emotionality items), and their interaction with Time, were added to our primary model (without MADRS) to determine if the effects of baseline Positive Emotionality remained after controlling for baseline extraversion. Analyses (which controlled for baseline LSAS and baseline Positive Emotionality, as well as DCS treatment condition) showed that baseline extraversion did not moderate the change in LSAS over time (p = .971), nor was it significantly related to LSAS scores at the 3-month follow-up (p = .612). BIC for this model was 8477.46, substantially higher than the BIC for the primary model (8463.97). The effects of baseline Positive Emotionality on LSAS remained significant, and decreased only slightly, *b* = −1.11, *95% CI* [−1.83, −0.41], *t*(1100) = −3.09, *p* = .002, *d* = 0.19, for the baseline Positive Emotionality × Time interaction, and *b* = −7.66, *95% CI* [−11.40, −3.92], *t*(1100) = −4.02, *p* < .001, *d* = 0.24, for the main effect of baseline Positive Emotionality on LSAS symptoms at the 3-month follow up.

### 2.3.3. Controlling for both depression and extraversion

Our final exploratory analysis added both baseline depression and baseline extraversion (excluding the 4 Positive Emotionality items), and their interactions with Time, to the primary model. In this analysis, baseline depression significantly moderated the change in LSAS over time, with participants who were higher on baseline depression improving less than those who were lower, *b* = 0.09, *95% CI* [0.05, 0.14], *t*(1087) = 4.07, *p* < .001, *d* = 0.25, and participants with higher baseline depression having higher LSAS scores at the 3-month follow-up, *b* = .61, *95% CI* [0.40, 0.82], *t*(1087) = 5.70, *p* < .001, *d* = 0.35. Extraversion, however, was not related to LSAS (ps > .163). Despite controlling for both of these potential confounders, the effects of baseline Positive Emotionality remained significant, *b* = −0.87, *95% CI* [−1.57, −0.16], *t*(1087) = −2.41, *p* = .016, *d* = 0.15, for the baseline Positive Emotionality × Time interaction, and *b* = −0.6.05, *95% CI* [−9.74, −2.37], *t*(1087) = −3.23, *p* = .001, *d* = 0.20, for the main effect of baseline Positive Emotionality on LSAS symptoms at the 3-month follow up. The BIC for this model was 8368.37, better than the BIC for the primary model (8463.97), but somewhat worse than the previous model which only added MADRS as an additional control variable.

In a further post-hoc analysis we found no evidence for an interaction between baseline depression and baseline Positive Emotionality affecting either change in LSAS over time (*p* = .125) or affecting LSAS scores at the 3-month follow-up (*p* = .332). The BIC for this model was 8505.37, substantially worse than the BIC for the primary model (8463.97).

Final post-hoc analyses were run because baseline MADRS scores were skewed, skewness = 1.14, with one outlier score of 43 (see eFig. 2c). This is not surprising given that most MARS scores were low because our inclusion criteria did not require participants to have elevated MADRS scores. Thus, the MLM analyses were rerun using MADRS scores that were square root transformed (skewness = 0.36, no outliers after transformation). Results for all analyses were identical (in terms of significance) using the square root transformed MADRS scores as with the raw MADRS scores. For example, in the exploratory analysis in which we controlled for MADRS scores (our first exploratory analysis reported above), higher transformed MADRS scores were still related to slower improvement in LSAS scores over time (p < .001 for the transformed MADRS × Time interaction), and higher Positive Emotionality scores were related to greater improvement over time (p < .001 for the Positive Emotionality × Time interaction). BIC for this model using the square root of MADRS (8360.78) was very similar to the BIC for the same model above using raw MADRS (8362.21).

## 3. Discussion

We investigated the relationship between baseline positive emotionality and treatment outcomes in adults with social anxiety disorder (SAD) who received exposure therapy as part of a d-cycloserine (DCS) augmentation trial (Smits et al., 2020). Higher positive emotionality was associated with superior treatment response – reflected in a steeper rate of decline in LSAS scores from before treatment to the 3-month follow-up, as well as lower symptom severity at 3-month follow-up. Results were robust when accounting for baseline depression severity and when accounting for extraversion (without the positive emotionality facet). These findings add to a growing literature supporting the potential value of positive emotions for improving treatment response for anxiety disorders.

Multiple lines of observational and experimental evidence support the premise that positive emotions may facilitate response to exposure-based therapies for anxiety (for a review, see (Taylor, Hoffman, & Khan, 2022)). Although the current study cannot answer *how* positive emotionality facilitated treatment success in patients receiving exposure therapy for SAD, extant studies point to several candidate mechanisms, including diminished threat reactivity, increased approach behaviors, distress tolerance, cognitive reappraisal, and inhibitory learning. That is, positive emotions may have increased behavioral engagement (Berman et al., 2019) and/or reduced subjective distress during exposure to perceived threat (i.e., public speaking exposures; (Taylor et al., 2020)), and may have promoted learning that the situation was less dangerous than initially expected (Zbozinek et al., 2015). Research is needed in which these and other putative mechanisms underpinning the positive emotion-exposure response link are assessed.

The current results are consistent with a prior study in which positive emotionality (similarly assessed using a personality inventory) predicted superior response in patients with GAD or panic disorder completing 10-sessions of CBT (Taylor, Knapp, et al., 2017); however, they differ from a 12-session CBT trial in SAD in which positive emotions were assessed in reference to participants’ experiences during the past week (Sewart et al., 2019). This divergence may be explained in part by the relative trait vs. state nature of the positive emotion assessments. To the extent that positive emotions experienced shortly before and throughout exposure exercises facilitated treatment response, the greater temporal stability of the personality-based positive emotion assessments at baseline may have better captured how participants were likely feeling at any given moment during treatment, including when completing exposure exercises. Future research could resolve this issue by examining state affect immediately before, during and after exposure exercises, alongside trait-based measures at baseline. Such data could also inform the optimal timing of positive emotion targeted augmentation strategies intended to boost exposure therapy efficacy. It should also be noted that the scope of the 4-item NEO-FFI positive emotionality assessment was limited compared to prior work (Sewart et al.). Research is needed to determine whether a broader measure of trait positive emotionality would yield similar findings or whether certain facets of positive emotionality (e.g., cheerfulness as assessed by the NEO-FFI) more strongly predict exposure therapy response relative to other facets.

Two exploratory outcomes are worth noting. First, non-positive emotionality facets of extraversion (sociability and activity) did not predict treatment response. This finding is consistent with prior work (Taylor, Knapp, et al., 2017) and suggests positive emotionality may serve unique functions in facilitating exposure response beyond general extraversion and its associated outcomes (e.g., positive social interactions). Second, baseline depression severity significantly moderated the change in LSAS over time, such that participants with higher depression scores improved less. This outcome suggests that features of depression beyond (low) positive emotionality interfered with treatment response. For example, sleep disturbance is common in those experiencing depression and was found to predict worse response to CBT for SAD (Zalta et al., 2013). Other candidate symptoms include diminished energy (which may limit treatment engagement) and concentration difficulties (which may impair memory for treatment content and/or directly interfere with extinction learning). Regardless, the current findings support positive emotionality as a strong predictor of response to exposure therapy for SAD, independent of variance shared with depression.

Clinical significance of the observed outcomes is reflected in the 44% larger reduction in symptoms experienced by participants scoring approximately one standard deviation above the sample mean on positive emotionality (score = 4) compared to those scoring one standard deviation below the mean (score = 2). The end state social anxiety scores of the low positive emotionality group remained above the LSAS cutoff of 60 typically used for identifying people who are eligible for entry into clinical trials and who are characterized by fear and avoidance of most social situations (reflecting the former DSM generalized subtype of social anxiety disorder; (Mennin et al., 2002)). The significance of these findings is further underscored by the prevalence of low positive emotionality in SAD samples. For example, nearly half (43%) of a sample of over 700 patients with SAD were characterized by low positive temperament (Tung & Brown, 2020), pointing to a sizable portion of the SAD population who may not respond sufficiently to first-line exposure-based therapies. The present findings suggest that directly targeting positive emotions before and/or while patients engage in exposure therapy may be a fruitful approach to improving treatment response – especially for people characterized by low positive emotionality. Accumulating evidence supports the initial efficacy of several cognitive and behavioral strategies focused on upregulating positive emotions in anxious populations, including acts of kindness (Alden & Trew, 2013), savoring (LaFreniere & Newman, 2023), and multicomponent protocols comprised of those and other (e.g., gratitude) activities (Craske et al., 2019; Taylor, Lyubomirsky, & Stein, 2017); however, to our knowledge, such approaches have not been directly tested in conjunction with exposure therapy. Although it remains to be established whether certain positive emotions facilitate exposure response more so than others, prior work suggests it is possible to increase a range of discrete positive emotions in anxious samples (Taylor, Lyubomirsky, & Stein, 2017), including those primarily measured by the NEO-FFI.

The following caveats should be considered when interpreting the study outcomes. Positive emotionality was measured before treatment using a self-report personality scale. It is not possible to determine whether positive emotions experienced throughout treatment or prior to exposure exercises accounted for the observed pattern of treatment response. Different positive emotions serve different functions (Shiota et al., 2017), suggesting it may be valuable to determine whether certain discrete positive emotions facilitate response better than others. Positive emotions were not manipulated in this study and therefore causality cannot be inferred. Finally, patients received five sessions of exposure therapy and it remains to be established whether positive emotionality predicts response to treatments of different durations and over the longer term (i.e., beyond three months following treatment cessation). Limitations notwithstanding, the current findings add to a growing literature supporting the value of assessing and possibly targeting positive emotions in treatment. Research is needed to identify mechanisms underlying the relationship between positive emotionality and treatment outcomes, and to identify whether, how, and when targeting positive emotions in the context of exposure therapy can optimize recovery from anxiety.

## Supplementary Material

Supplement

## Data Availability

Data will be made available on request.
