# Supplementary material for: What good are positive emotions for treatment? A replication test of whether trait positive emotionality predicts response to exposure therapy for social anxiety disorder
Source: Behav Res Ther. Author manuscript; Available in PMC 2024 Dec 1. (PMC10862259; doi:10.1016/j.brat.2023.104436)
Supplement: Supplement [file NIHMS1958883-supplement-Supplement.docx]

Supplemental Materials

Covariance Structures for the Error Covariance Matrix

In the present investigation, there were 8 repeated assessments over time for each participant, and hence there was an error in predicting the outcome at each of these 8 assessments. The error covariance matrix is the 8 x 8 covariance matrix for the variances and covariances of these errors at the 8 assessment points. The unstructured covariance matrix freely estimates each variance and covariance in this covariance matrix. The diagonal matrix freely estimates a different variance at each timepoint, but assumes that the covariances are all 0. AR(1) assumes that the variances at the 8 timepoints are homogeneous, and that the covariances between adjacent assessments (e.g., assessment 1 with assessment 2; assessment 2 with 3, etc.) are all the same (ρ). Assessments that are 2 timepoints apart are assumed to have a covariance of ρ^2^. Assessments that are 3 timepoints apart have a covariance of ρ^3^, and so on. Compound symmetry assumes homogeneous variances (like AR(1)), but assumes that all the covariances are equal, regardless of their separation in time. Toeplitz constrains all variances to be homogeneous and constrains each covariance that is separated by the same number of timepoints to be equal (i.e., the covariance between assessments 1 and 3 is constrained to be the same as the covariance between assessments 2 and 4, and between 3 and 5). We ran our MLM models multiple times, each using a different covariance structure. We also ran the MLM models using random effects models to account for the covariance of the errors of the repeated measures (Hoffman, 2016). We selected as our final model the one with the covariance structure that best fit the data (i.e., the structure that generated lowest BIC). That covariance structure was AR(1).

eTable 1. Sample characteristics at Baseline

| Treatment Condition: | **Pre-session (n = 38)** | **Post-session (n = 36)** | **Tailored  (n = 40)** | **Placebo (n = 38)** |
| --- | --- | --- | --- | --- |
|  | N or M (% or SD) | N or M (% or SD) | N or M (% or SD) | N or M (% or SD) |
| Age | 29.73 (10.42) | 27.54 (8.29) | 30.73 (9.88) | 28.76 (11.81) |
| Sex |  |  |  |  |
| Male | 16 (42.11) | 15 (41.67) | 20 (50) | 16 (42.11) |
| Female | 22 (57.89) | 21 (58.33) | 19 (47.5) | 22 (57.89) |
| genderqueer | 0 (0) | 0 (0) | 1 (2.5) | 0 (0) |
| Race |  |  |  |  |
| White | 23 (57.5) | 18 (45) | 25 (62.5) | 24 (60) |
| Black or African American | 5 (12.5) | 3 (7.5) | 8 (20) | 4 (10) |
| Asian | 7 (17.5) | 13 (32.5) | 6 (18) | 8 (20) |
| Other | 1 (2.5) | 2 (5) | 0 (0) | 2 (5) |
| Not Reported | 2 (5) | 0 (0) | 1 (2.5) | 0 (0) |
| Ethnicity |  |  |  |  |
| Not Hispanic/Latino | 32 (84.21) | 25 (69.44) | 33 (82.5) | 26 (68.42) |
| Hispanic/Latino | 6 (15.79) | 9 (25) | 5 (12.5) | 10 (26.32) |
| Not reported | 0 (0) | 2 (5.56) | 2 (5) | 2 (5.26) |
| Marital Status |  |  |  |  |
| Single | 27 (71.05) | 30 (83.33) | 27 (67.5) | 27 (71.05) |
| Living with partner | 3 (7.89) | 2 (5.56) | 4 (10) | 6 (15.79) |
| Married | 8 (21.05) | 3 (8.33) | 7 (17.5) | 4 (10.53) |
| Divorced | 0 (0) | 1 (2.78) | 2 (5) | 1 (2.63) |
| Highest Education |  |  |  |  |
| Graduate School | 14 (36.84) | 13 (36.11) | 18 (45) | 5 (13.16) |
| College Graduate | 14 (36.84) | 9 (25) | 12 (30) | 17 (44.74) |
| Partial College | 8 (21.05) | 13 (36.11) | 9 (22.5) | 14 (36.84) |
| High School Graduate | 1 (2.63) | 1 (2.78) | 1 (2.5) | 2 (5.26) |
| Partial High School | 1 (2.63) | 0 (0) | 0 (0) | 0 (0) |
| Highest Occupation |  |  |  |  |
| Executive | 0 (0) | 1 (2.78) | 1 (2.5) | 0 (0) |
| Manager/Professional | 15 (39.47) | 13 (36.11) | 15 (37.5) | 9 (23.68) |
| Administrative | 5 (13.16) | 5 (13.89) | 5 (12.5) | 7 (18.42) |
| Clerical | 3 (7.89) | 1 (2.78) | 1 (2.5) | 7 (18.42) |
| Skilled | 9 (23.68) | 5 (13.89) | 7 (17.5) | 7 (18.42) |
| Semi-Skilled | 3 (7.89) | 4 (11.11) | 8 (20) | 6 (15.79) |
| Unskilled | 2 (5.26) | 4 (11.11) | 1 (2.5) | 0 (0) |
| Never Worked | 1 (2.63) | 3 (8.33) | 2 (5) | 2 (5.26) |
| Living Situation |  |  |  |  |
| Urban | 28 (73.68) | 30 (83.33) | 29 (72.5) | 29 (76.32) |
| Suburban | 8 (21.05) | 6 (16.67) | 11 (27.5) | 8 (21.05) |
| Rural | 2 (5.26) | 0 (0) | 0 (0) | 1 (2.63) |
| Income |  |  |  |  |
| Not given | 8 (21.05) | 4 (11.11) | 7 (17.5) | 9 (23.68) |
| $0 to $4,999 | 3 (7.89) | 10 (27.78) | 4 (10) | 4 (10.53) |
| $5,000 to $9,999 | 2 (5.26) | 1 (2.78) | 2 (5) | 1 (2.63) |
| $10,000 to $14,999 | 2 (5.26) | 2 (5.56) | 1 (2.5) | 4 (10.53) |
| $15,000 to $24,999 | 2 (5.26) | 3 (8.33) | 5 (12.5) | 5 (13.16) |
| $25,000 to $34,999 | 5 (13.16) | 4 (11.11) | 2 (5) | 3 (7.89) |
| $35,000 to $49,999 | 5 (13.16) | 0 (0) | 6 (15) | 6 (15.79) |
| $50,000 to $74,999 | 9 (23.68) | 6 (16.67) | 7 (17.5) | 2 (5.26) |
| > $75,000 | 2 (5.26) | 6 (16.67) | 6 (15) | 4 (10.53) |
| Occupational Status |  |  |  |  |
| Not Applicable | 2 (5.26) | 5 (13.89) | 3 (7.5) | 7 (18.42) |
| Full-time employment | 20 (52.63) | 15 (41.67) | 19 (47.5) | 19 (50) |
| Part-time employment | 7 (18.42) | 6 (16.67) | 6 (15) | 5 (13.16) |
| Dependent on spouse or is a student | 9 (23.68) | 10 (27.78) | 12 (30) | 7 (18.42) |
| Baseline Clinical Characteristics |  |  |  |  |
| LSAS | 80.61 (14.73) | 86.25 (18.43) | 85.65 (15.05) | 85.24 (16.03) |

Note. LSAS = Liebowitz Social Anxiety Scale. The 4 treatment conditions reflect the timing of the d-cycloserine dose.eFIgure 1. CONSORT Flow Diagram from Parent Trial (NCT02066792)


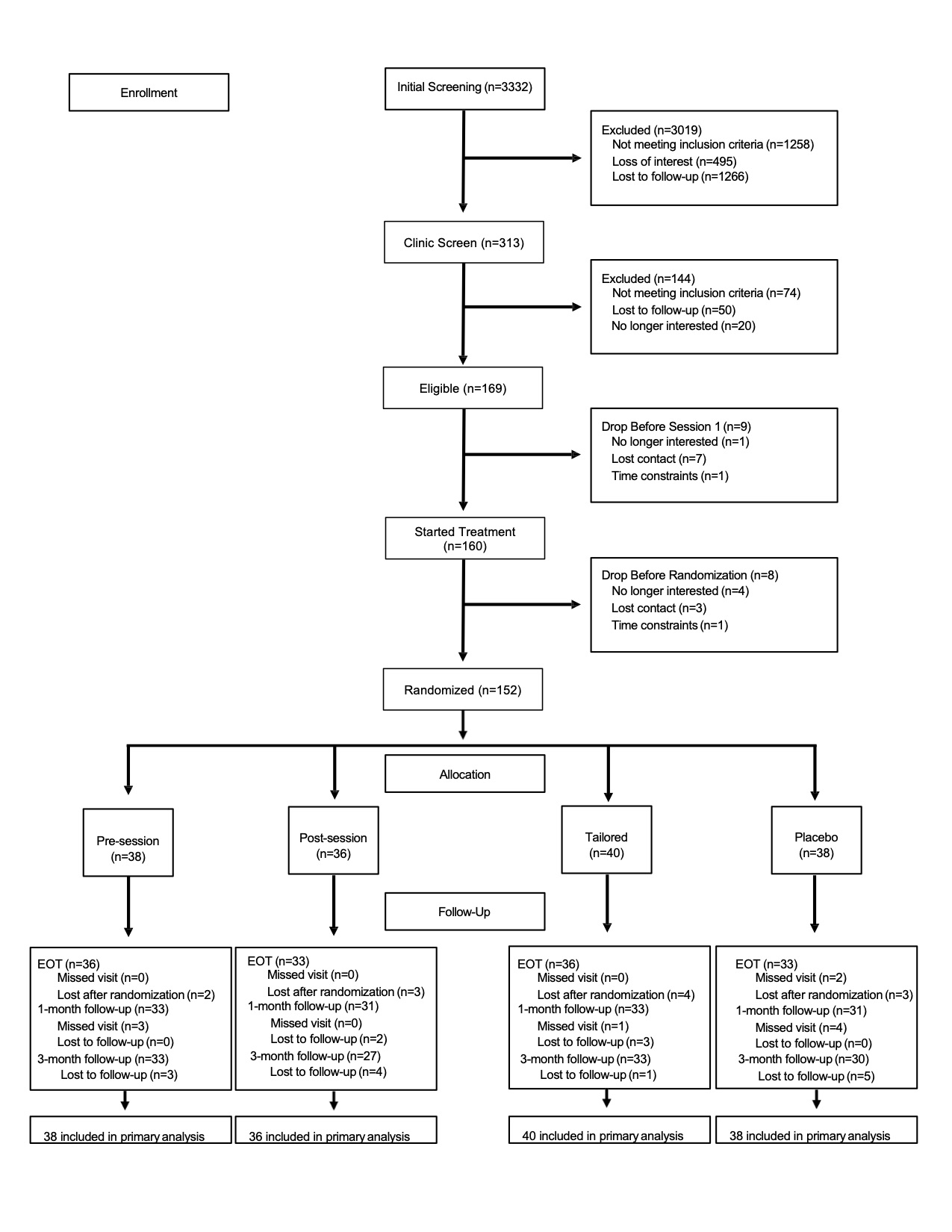


Note. EOT = end of treatment (1-week follow-up).

eFIgure 2. Distributions of the Variables of Interest

2a. LSAS (possible range: 0-144)

2b. Positive Emotionality (possible range: 1-5)


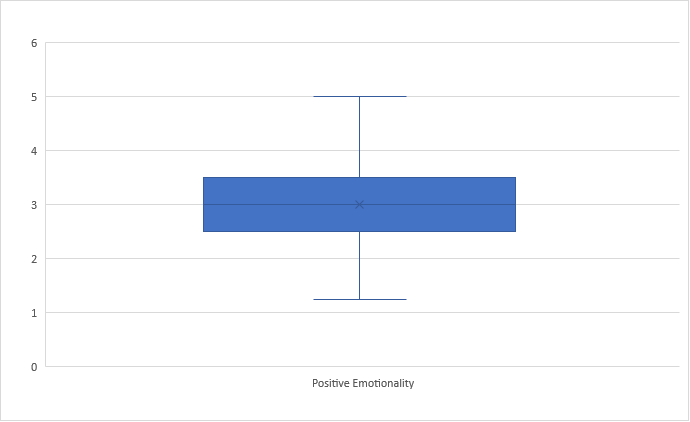


2c. MADRS (possible range: 0-60)


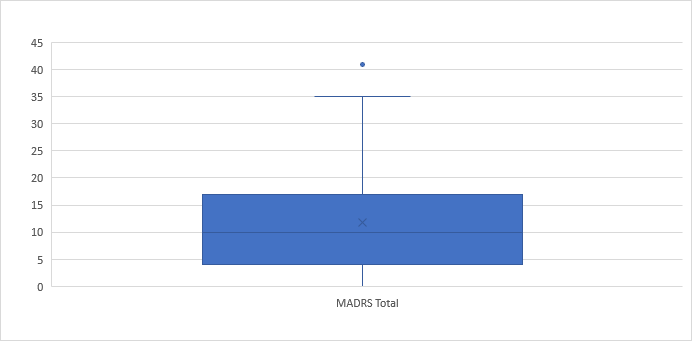


2d. Extraversion without Positive Emotionality Items (possible range: 1-5)


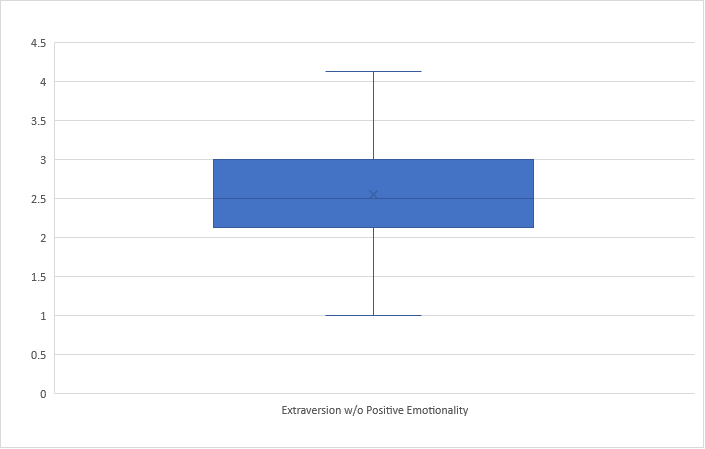


eFIgure 3. Scatterplots for the Relation between Baseline scores on LSAS (social anxiety), Positive Emotionality, MADRS (depressive symptoms), and Extraversion without Positive Emotionality.

Note: LSAS and MADRS scores are total scores for the full scales. Positive Emotionality and Extraversion without Positive Emotionality were scales created for this investigation and hence, these scores reflect average score per item on the subscale (range: 1-5) rather than total scores.

3a. Baseline LSAS with Positive Emotionality

3b. Baseline LSAS with MADRS (depression)

3c. Baseline LSAS with Extraversion without Positive Emotionality

3d. Baseline Positive Emotionality with MADRS

3e. Baseline Positive Emotionality with Extraversion without Positive Emotionality

3f. Baseline MADRS with Extroversion without Positive Emotionality

eFigure 4. Spaghetti plots for a subset of the total sample.

Note: Figure 4a shows the spaghetti plots for the first 15 participants who were classified as “low Positive Emotionality” (in the bottom 25^th^ percentile). Figure 4b shows the spaghetti plots for the first 15 participants classified as “high Positive Emotionality” (in the top 25^th^ percentile).

*4a.*


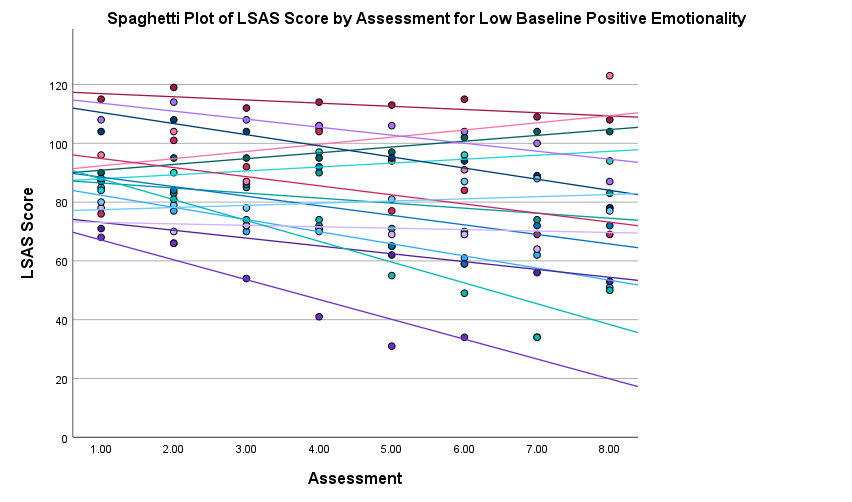


4b.


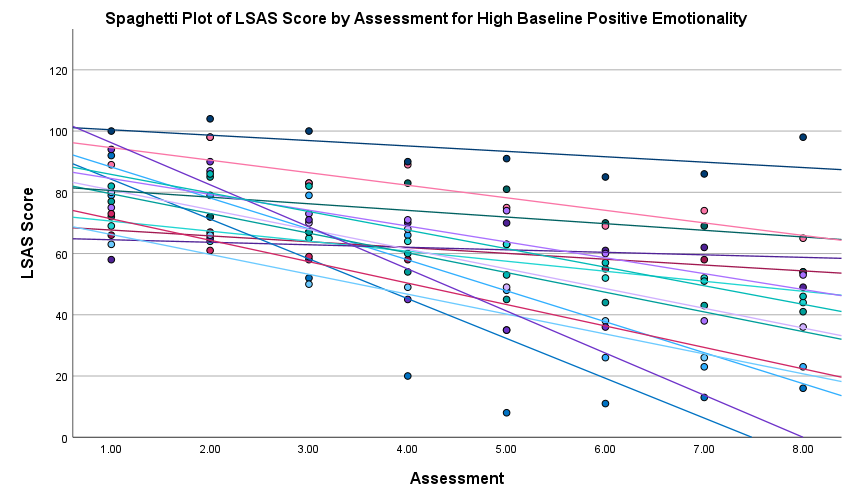


eFigure 5. Estimated LSAS scores from baseline through 3-month follow-up at different levels of baseline MADRS depression severity scores.

Note: 1MFU = 1-month follow-up; 3MFU = 3-month follow-up. Low MADRS represent participants with scores in the bottom 25^th^ percentile for this sample (4 or less), while High MADRS represent participants with scores in the upper 25^th^ percentile for this sample (17 or greater). These estimated means come from analyses where baseline LSAS and baseline Positive Emotionality, and their interactions with Time, are controlled.
